# MSCs-Derived Exosomes and Neuroinflammation, Neurogenesis and Therapy of Traumatic Brain Injury

**DOI:** 10.3389/fncel.2017.00055

**Published:** 2017-02-28

**Authors:** Yongxiang Yang, Yuqin Ye, Xinhong Su, Jun He, Wei Bai, Xiaosheng He

**Affiliations:** ^1^Department of Neurosurgery, Xijing Hospital, Fourth Military Medical UniversityXi’an, China; ^2^Department of Neurosurgery, PLA 422nd HospitalZhanjiang, China; ^3^Department of Neurosurgery, PLA 163rd Hospital (Second Affiliated Hospital of Hunan Normal University)Changsha, China

**Keywords:** exosomes, mesenchymal stem cells (MSCs), traumatic brain injury (TBI), neuroinflammation, neurogenic niches, neurogenesis, therapy

## Abstract

Exosomes are endosomal origin membrane-enclosed small vesicles (30–100 nm) that contain various molecular constituents including proteins, lipids, mRNAs and microRNAs. Accumulating studies demonstrated that exosomes initiated and regulated neuroinflammation, modified neurogenic niches and neurogenesis, and were even of potential significance in treating some neurological diseases. These tiny extracellular vesicles (EVs) can derive from some kinds of multipotent cells such as mesenchymal stem cells (MSCs) that have been confirmed to be a potentially promising therapy for traumatic brain injury (TBI) in experimental models and in preclinical studies. Nevertheless, subsequent studies demonstrated that the predominant mechanisms of MSCs’s contributions to brain tissue repairment and functional recovery after TBI were not the cell replacement effects but likely the secretion-based paracrine effects produced by EVs such as MSCs-derived exosomes. These nanosized exosomes derived from MSCs cannot proliferate, are easier to preserve and transfer and have lower immunogenicity, compared with transplanted exogenous MSCs. These reports revealed that MSCs-derived exosomes might promise to be a new and valuable therapeutic strategy for TBI than MSCs themselves. However, the concrete mechanisms involved in the positive effects induced by MSCs-derived exosomes in TBI are still ambiguous. In this review, we intend to explore the potential effects of MSCs-derived exosomes on neuroinflammation and neurogenesis in TBI and, especially, on therapy.

## Introduction

Exosomes are the smallest endocytic origin membrane-bound nanovesicles which participate in the complex intercellular communication system (Lamichhane et al., 2015; Merino-Gonzalez et al., 2016). They are released from various cell types under normal or pathological conditions, influencing the activity of recipient cells by carrying active signals. Exosomes contain not only cellular proteins and lipids, but also messenger RNAs (mRNAs) and microRNAs (miRNAs) from the host cell (Barteneva et al., 2013). The potential clinical values of exosomes can be attributed to their characteristics such as: their surface markers and molecular cargoes that can be taken as potential diagnostic biomarkers for some diseases (Muller, 2012; Dorayappan et al., 2016; Escudero et al., 2016), their low immunogenicity and long half-life in circulation and their ability to cross the brain-blood barrier (BBB) (Sun et al., 2010; Alvarez-Erviti et al., 2011; Kalani et al., 2014), and their potential roles as mediators of the regenerative responses (Lai et al., 2010; Xin et al., 2012). As for the relationships between exosomes and neurological diseases, the cell-based therapy such as stem cell therapy should be mentioned first. At present, stem cell therapy has been proposed to be a promising therapeutic option for several neurological disorders including Stroke, Parkinson’s disease, Amyotrophic Lateral Sclerosis and Huntington’s disease (Yoo et al., 2013). As the secretomes of the stem cells appear to be of greater benefit on tissue regeneration and repair than the stem cells themselves (Teixeira et al., 2013), the extracellular components such as stem cell-derived exosomes that are responsible for their therapeutic benefits have begun to be noticed. One of the most popular stem cell-derived exosomes which are widely applied in neurological diseases researches are the mesenchymal stem cells (MSCs)-derived exosomes. It has been systematically elaborated in several latest reviews that MSCs-derived exosomes play an important role in neuroinflammation, neurogenic niches and neurogenesis, and therapeutic strategy of neurological diseases (Gupta and Pulliam, 2014; Batiz et al., 2015; Luarte et al., 2016; Marote et al., 2016).

Traumatic brain injury (TBI) is a set of secondary pathological and/or functional alteration within brain due to a sudden external force, a major cause of death and longterm disability worldwide (Menon et al., 2010; Chauhan, 2014). Until recently, no clinical trials have identified effective pharmacologic treatments in TBI (Narayan et al., 2002; Xiong et al., 2013; Skolnick et al., 2014; Wright et al., 2014). In the past decade, lots of studies demonstrated that MSCs had promised to be an effective therapy for brain injury not only in experimental models of TBI (Lu et al., 2001; Chopp and Li, 2002; Mahmood et al., 2004a,b; Li and Chopp, 2009; Nichols et al., 2013), but also potentially in clinical practices (Zhang et al., 2008; Cox et al., 2011). However, the dominating mechanisms of MSCs’s participation in brain tissue repairment and functional restoration after TBI are likely related to their secretion-based paracrine effects rather than the cell replacement effects (Chopp and Li, 2002; Li and Chopp, 2009). One sort of the MSCs secretion-based paracrines are MSCs-derived exosomes that characterized by without proliferation, less immunogenic and easier to store and deliver than MSCs (Lai et al., 2011). Newly studies indicate that MSCs-derived exosomes can improve functional recovery, rescue pattern separation and spatial learning impairments, promote neurovascular remodeling (neurogenesis and angiogenesis) and reduce neuroinflammation in animal models after TBI (Zhang et al., 2015; Kim et al., 2016; Zhang Y. et al., 2016). Although the above reports reveal that MSCs-derived exosomes may promise to be a better therapy for TBI than MSCs, the concrete mechanisms and molecular constituents that involved are still not clear. In order to address this issue preliminarily, we proposed this review to introduce the foremost new progresses in paracrine mechanisms of MSCs-derived exosomes and explore their potential effects on neuroinflammation, neurogenesis, and especially, on therapy of TBI.

## Exosomes

The extracellular vesicles (EVs) have been recently identified as a novel type of mediators in intercellular communication. Although EVs were initially regarded as membrane-enclosed vesicles secreted just by outward shedding or budding of the cells’ plasma membrane (PM), it is recently known that various types of EVs with different formation approaches coexist in the extracellular milieu including different tissues and biological fluids (Colombo et al., 2014). Some types are originated from shedding of the PM, while others are secreted by exocytosis after fusion of the lumen that is so-called multivesicular bodies (MVBs) or multivesicular endosomes with the PM (Colombo et al., 2014). Based on these various intracellular origins and biogenesis formation approaches, EVs may have different compositions, functions and types (Kowal et al., 2014). Exosomes are thought to be one subtype of the most thoroughly studied EVs which are formed inward MVBs through the endolysosomal approach and are released from fusion of MVBs with the PM (Kalani et al., 2014). They are membrane-enclosed vesicles and contain various molecular constituents including cell type characteristic combination of lipids, proteins, coding and non-coding RNAs (Lener et al., 2015). In recent years, a lot of researches about exosomes with the purpose to explore the primary mechanisms of their formation and secretion and their roles in intercellular communication have been conducted and made great progresses. The main characteristics of exosomes were summarized in **Table 1**.

**Table 1:** The characteristics of exosomes.

Characteristics	Description
Definition	The smallest endocytic origin membrane-bound nanovesicles
Size	30–100 nm
Cellular origin	Various cell types under normal or pathological conditions
Formation mechanisms	Forming MVBs by the ESCRT dependent and ESCRT independent approaches
Main contents	Proteins, lipids, mRNAs and miRNAs from the host cells
Membrane markers	CD9, CD63, CD81, CD82
Biogenesis functions	Mediate diverse functions in intercellular communication
Main advantages	Low immunogenicity, long half-life in circulation and ability to cross the BBB


*MVBs, multivesicular bodies; ESCRT, Endosomal Sorting Complex Required for Transport; mRNAs, messenger RNAs; miRNAs, micro RNAs; BBB, brain-blood barrier.*

### Biogenesis of Exosomes

Exosomes are 30–100 nm endocytic origin membrane vesicles secreted by various types of cells and mediate diverse functions in intercellular communication (Lener et al., 2015; Willms et al., 2016). As a well characterized population of EVs, exosomes can be distinguished by the combination of their size and origin. They originate in the endocytic approaches which are involved in the trafficking of several proteins that are internalized and can either recycle back to the PM or get sorted to degradation (Gould and Lippincott-Schwartz, 2009; Klumperman and Raposo, 2014). In these approaches, early endosomes develop into late endosomes, and they experience an inward budding procedure of their membrane and form the intraluminal vesicles (ILVs) with diameter ranging approximately from 30 to 100 nm during the developing process. The late endosomes, often termed MVBs because of containing ILVs, may either fuse with the lysosome and lead their contents to be degraded, or fuse with the PM that permits the ILVs to be discharged into the extracellular environment, these EVs are then defined as exosomes (van Niel et al., 2006). The formation of ILVs and exosomes is a modulated procedure that includes arranging endosomal membrane into distinctive domains which are extremely rich in special sets of membrane proteins. One set of proteins include CD81, CD82, CD63, and CD9 which are intensively assembled on the exosome membranes and act as marker proteins for these vesicles. The other set of proteins are the components of the Endosomal Sorting Complex Required for Transport (ESCRT) equipment that are approximately constituted by thirty proteins which form four complexes (ESCRT-0, ESCRT-I, ESCRT-II, ESCRT-III) (Hanson and Cashikar, 2012). As for the mechanism of the formation of MVBs and ILVs, there are two main pathways that have been described: the ESCRT dependent and ESCRT independent mechanisms. The ESCRT dependent mechanism is the best-described pathway that sequesters ubiquitinated membrane proteins into an endosomal microdomain and induces an invagination that generates ILVs formation harboring this cargo. ESCRT-0 complex acts a crucial role in determining the outcome of membrane proteins enriched on endosomes, it identifies and secludes ubiquitinated transmembrane proteins on the endosomal membrane and further separates them into microdomains and combines the ESCRT-I complex. ESCRT-I complex, the first described ESCRT complex, is essential for sorting cargo into the MVBs and efficiently creating ILVs. It recruits ESCRT-II complex after binding ESCRT-0 complex in turn, and then both complexes initiate membrane deformation into buds with sorted cargo that allow cytosolic proteins and RNAs (mRNAs and miRNAs) to get into the forming vesicles. ESCRT-II connects ESCRT-I and ESCRT-III and works in the canonical ESCRT pathway. ESCRT-III differs from the other ESCRT complexes in that its components are soluble monomeric proteins that are related to each other and polymerize into filaments when recruited to the membrane, and activated by interaction with upstream pathway components. The ESCRT-II complex first recruits ESCRT-III subunits inside nascent vesicles and then together with the ATPase VPS4 provokes the membrane cleavage process required to free ILVs, during which ESCRT subunits and ubiquitin molecules are secreted into the cytosol for further recycling (Hanson and Cashikar, 2012). The exosomes biogenesis also occurs potentially via ESCRT-independent mechanisms which have been recently proposed as alternative pathways for the formation and assembling special cargo into exosomes, involving a lipid-driven mechanism (Trajkovic et al., 2008) and a sphingosine 1-phosphate receptor dependent mechanism (Kajimoto et al., 2013). In addition, another alternative mechanism that only needs the enlisting of the ESCRT-III subgroup CHMP4 can also be a pathway for the formation and sorting of exosomes by interacting an ESCRT-III binding protein ALIX with proteins comprising of late-domain motif LYPXnL like the GPCR Par1 or syntenin (Sette et al., 2010; Baietti et al., 2012; Hurley and Odorizzi, 2012).

### Contents of Exosomes

Exosomes are lipid-bilayer vesicles that contain cellular specific lipids, proteins, coding and non-coding RNAs (Lener et al., 2015). The proteins contained in most exosomes include membrane proteins of the tetraspanin family (CD81, CD82, CD63, and CD9), proteins associated with MVB biogenesis (Alix, TSG101) that participates in the ESCRT, heat shock proteins (HCP/HSP 70 and 90) and proteins required for membrane transport and fusion (Rab GTPases, annexins, and flotillins) (Ohno et al., 2013; Raposo and Stoorvogel, 2013). In addition, cytoskeletal proteins (actin, syntenin, and moesin), signal transduction proteins (kinase proteins) and metabolic enzymes (GAPDH, LDHA, PGK1, aldolase, and PKM) are carried by exosomes as well (Chaput and Thery, 2011). The lipids compositions contained in exosomes usually exist on the exosomal membrane where there are numerous lipid-rafts consisting of cholesterol, ceramide, sphingomyelin and glycerophospholipids with long and saturated fatty-acyl chains (Thery et al., 2006; Kowal et al., 2014). The coding and non-coding RNAs contained in exosomes generally refer to mRNAs and miRNAs. The mRNAs enclosed in exosomes may play more of a regulatory rather than a functional role (Batagov and Kurochkin, 2013), though they can be readily translated to proteins by the recipient cells (Valadi et al., 2007). The miRNAs are the most widely described ncRNAs of exosomes that target the 3′ untranslated region (UTR) of specific mRNAs to inhibit their translation in most cases. As a consequence of this, miRNAs can modify the phenotype and/or the physiology of the recipient cell, modulating cellular processes as relevant as proliferation, differentiation and survival among others (Cocucci and Meldolesi, 2015). Although all exosomes contain the constitutive array of proteins, lipids and RNAs as illustrated above, their contents vary in accordance with the cellular origin and the physiological or pathological condition of the cell and its extracellular environment. Therefore, the contents of exosomes not only relate to their different cellular functions, but also act as indicators of their cellular sources. Furthermore, the distinct exosomes that enclose different proteins and RNA compositions determine their various subpopulations and, therefore, yield different effects on recipient cells.

## MSCs-Derived Exosomes

Mesenchymal stem cells-derived exosomes are one of the most popular stem cell-derived exosomes that are widely applied in neurological diseases researches. MSCs are a sort of adult multipotent cells owning the self-renew capacity and differentiation potential which have been investigated in cell replacement strategies with the purpose to repair damaged adult mesenchymal or ectodermal tissues such as neural tissue (Pittenger et al., 1999; Donega et al., 2014; Takeda and Xu, 2015; Bagher et al., 2016). However, the primary mechanism of MSCs mediated regeneration effect is not its cellular engraftment and differentiation but a secretome-based paracrine activity. One of the bioactive factors secreted from MSCs is the MSCs-derived exosomes which were firstly isolated and characterized from human ESC-derived MSCs conditioned medium in Lai et al. (2010). Compared to other cell types, the MSC is the most prolific exosome producer that can produce large amounts of exosomes which are not different from the exosomes derived from other sources in terms of morphological features, isolation and storage conditions (Yeo et al., 2013). As the constituents of exosomes are dependent on their cellular origin (Raposo and Stoorvogel, 2013), the contents of MSCs-derived exosomes have their own characteristics. In addition to the common surface markers of exosomes, MSCs-derived exosomes involve special membrane binding proteins such as CD73, CD44, and CD29 (Lai et al., 2012). The protein components of MSCs-derived exosomes also changed when these cells were obtained from different MSCs cultured media. RNAs such as mRNA and miRNAs are encapsulated in MSCs-derived exosomes as well. Most of the studies have demonstrated that MSCs-derived exosomes contained various miRNAs which participated in the cell–cell communication and altered the fate of recipient cells (Koh et al., 2010; Xin et al., 2012, 2013; Katakowski et al., 2013; Lee et al., 2013; Ono et al., 2014). It has been widely accepted that the exosome secretion is an efficient adaptive mechanism that environmental challenges can influence the composition, biogenesis, and secretion of exosomes. The MSCs-derived exosomes profile can be modified by pretreatment as well. One research indicated that the amount of miR-133b in MSCs and their released exosomes significantly elevated when MSCs were *in vitro* exposed to brain tissue extracted from rats suffered from middle cerebral artery occlusion (MCAo), compared to MSCs which were exposed to normal brain tissue extracts (Xin et al., 2012). Another research also indicated that the miR-22 level in MSCs-derived exosomes increased after ischemic preconditioning (Feng et al., 2014). Above researches indicate that the MSC and its environment can deliver feedback information to each other through MSCs-derived exosomes. That is to say, the environment of MSC will modify the contents of its secreted exosomes which can affect and modify the tissue environment consequently.

In recent years, a plenty of researches have been conducted to explore the potential therapeutic values of MSCs-derived exosomes from different MSCs sources. The most common MSCs sources are bone marrow (BM) (Zhu et al., 2012; Zhang Y. et al., 2016), embryonic stem cell (ESC) (Lai et al., 2010; Zhang et al., 2014; Zhang S. et al., 2016) and umbilical cord (UC) (Fang et al., 2016; Zhang B. et al., 2016), and others include adipose (AD) (Hu et al., 2016), induced pluripotent stem cell (iPSC) (Qi et al., 2016), menstrual (Men) (Lopez-Verrilli et al., 2016) and synovial (Guo et al., 2016). Accordingly, exosomes derived from different sources MSCs might have different isolation, identification and delivery methods. Ultracentrifugation is the most common method used to isolate exosomes by which the exosomes are precipitated by centrifugation at ≥100,000 × *g* for at least 1 h. Other methods include high-performance liquid chromatography (HPLC), ultrafiltration and ExoQuick-TC. Common methods used to identify exosomes include Electron microscopy for the morphology identification, Flowcytometry and Western blotting for the membrane marker (CD9, CD63, and CD81) identification. The exosomes can be quantified by measuring the total protein concentration using BCA protein assay kit, ELISA kit and other kinds of kit. The concrete amounts of MSCs-derived exosomes that were obtained and used in the researches had great differences according to the MSCs sources, isolation and quantification methods and experiment protocols. Methods used to delivery exosomes include intravenous/subcutaneous/intra-articular injection *in vivo* and coculture *in vitro*. Though many previous studies demonstrated various methods for the isolation, identification and delivery of MSCs-derived exosomes, there is no standard consensus until now. The different isolation, identification and delivery methods of various human origin MSCs derived exosomes were summarized in **Table 2**.

**Table 2:** The different isolation, identification and delivery methods of various origin MSC derived exosomes.

Origin	Isolation method	Identify method	Delivery method	Reference
hBM-MSC	Ultracentrifugation (100,000 × *g* 1 h)/ultrafiltration/ExoQuick-TC	Western blotting	Subcutaneous/intravenous injection	Zhu et al., 2012;Zhang Y. et al., 2016
hESC-MSC	Ultracentrifugation (100,000 × *g* 1 h)/ultrafiltration/HPLC	Flowcytometry, Western blotting	Intravenous/intra-articular/subcutaneous injection	Lai et al., 2010; Zhang et al., 2014; Zhang S. et al., 2016
hUC-MSC	Ultracentrifugation (120,000 × *g* 70 min)/ (100,000 × g 1 h)	Electron microscopy, Western blotting	Subcutaneous injection, *in vitro* coculture	Fang et al., 2016; Zhang B. et al., 2016
hAD-MSC	Ultracentrifugation (120,000 × *g* 90 min)	Electron microscopy, Western blotting	Intravenous injection	Hu et al., 2016
hiPSC-MSC	Ultracentrifugation (100,000 × *g* 2 h)	Western blotting	Intravenous injection	Qi et al., 2016
hMen-MSC	Ultracentrifugation (100,000 × *g* 70 min)	Electron microscopy, Flowcytometry, Western blotting	*In vitro* coculture	Lopez-Verrilli et al., 2016
hS-MSC	Ultracentrifugation (100,000 × *g* 1 h)	Western blotting	Intravenous injection	Guo et al., 2016


*MSC, mesenchymal stem cell; hBM, human bone marrow; hESC, human embryonic stem cells; hUC, human umbilical cords; hAD, human adipose; hiPSC, human induced pluripotent stem cell; hMen, human menstrual; hS, human synovial; HPLC, high-performance liquid chromatography.*

## MSCs-Derived Exosomes and TBI

Previous studies indicated that MSCs promised to be an effective therapy for brain injury in experimental models and clinical practices of TBI (Lu et al., 2001; Chopp and Li, 2002; Mahmood et al., 2004a,b; Zhang et al., 2008; Li and Chopp, 2009; Cox et al., 2011; Nichols et al., 2013). However, it has been documented recently that the major mechanisms of MSCs participating in brain remodeling and functional recovery after TBI are not the cell replacement effects but likely the paracrine effects of secretion-based factors such as MSCs-derived exosomes (Chopp and Li, 2002; Li and Chopp, 2009; Lai et al., 2011). Excitingly, newly studies demonstrate that MSCs-derived exosomes can reduce neuroinflammation, promote neurogenesis and angiogenesis, rescue pattern separation and spatial learning impairments, and improve functional recovery after TBI in animal models (Zhang et al., 2015; Kim et al., 2016; Zhang Y. et al., 2016). These reports indicate that MSCs-derived exosomes may promise to be a better therapy for TBI than MSCs.

**FIGURE 1:**
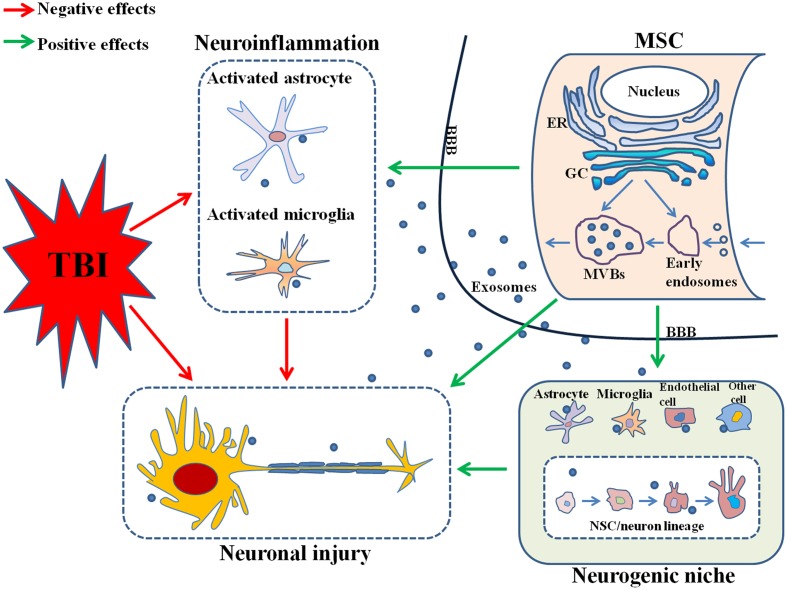
**Mesenchymal stem cells (MSCs)-derived exosomes reduce neuroinflammation by suppressing the activation of astrocytes and microglia and promote neurogenesis possibly by targeting the neurogenic niche, both which contribute to nervous tissue repair and functional recovery after TBI.** MSC, mesenchymal stem cell; TBI, traumatic brain injury; ER, endoplasmic reticulum; GC, Golgi complex; MVBs, multivesicular bodies; BBB, brain-blood barrier; NSC, neural stem cell.

In the above paragraphs, we have reviewed the biogenesis and contents of exosomes and MSCs-derived exosomes. And we will go on in the next paragraphs to further discuss the roles of MSCs-derived exosomes in the neuroinflammation, neurogenesis and therapy of TBI, the probable mechanisms and molecular constituents that might be involved in, and some preliminary suggestions related to future researches. The probable mechanisms of the effects produced by MSCs-derived exosomes on TBI were illustrated in **Figure 1**.

### MSCs-Derived Exosomes and the Neuroinflammation of TBI

Neuroinflammation plays an important role in the pathogenesis of TBI and occurs during its whole primary and secondary stages (Lozano et al., 2015). It seems to be responsible for not only the detrimental effects contributing to primary insult and secondary injury but also the beneficial effects facilitating tissue repair (Woodcock and Morganti-Kossmann, 2013). The development of neuroinflammation occurring within the brain following TBI involves a complex process of cumulative alterations. Instantly after the TBI insult, multiple types of quiescent glial cells become rapidly activated via a “reactive gliosis” procedure which includes initiating microglia activation and continuing astrocytic activation via the generation and secretion of inflammatory mediators which further work on surrounding neurons and glial cells in turn (Chiu et al., 2016). This brain inflammation reaction in TBI is featured by glial cells activation, leukocytes recruitment, and generation and release of inflammatory mediators such as chemokines and cytokines (Morganti-Kossmann et al., 2001). These inflammatory mediators not only influence surrounding glial cells and neurons but also recruit peripheral immune cells such as neutrophils, macrophages and lymphocytes into brain. Though the above cellular endogenous inflammatory responses triggered by the primary insult are aiming to repair the damaged tissue, the excessive production of pro-inflammatory cytokines usually become a significant driving force for the pathological progression of TBI. The excessive neuroinflammation not only initiates a primary damage phase which involves diffuse axonal injury, contusion and laceration, intracranial hemorrhage and brain swelling that invariably leads to instant cell death (LaPlaca et al., 2007; Cheng et al., 2012; Greig et al., 2014), but also induces an extended secondary phase that involves cascades of biological processes aiming to potentially restore the cellular homeostasis of the impaired brain tissue which is not particularly well controlled and often will result in exacerbation of the primary injury, progressive neurodegeneration and delayed cell death (Kabadi and Faden, 2014; Lozano et al., 2015). Herein, how to alleviate the acute neuroinflammation following an initial TBI insult and further beneficially establish new therapies for TBI is a hot research topic.

Recently, two studies have demonstrated that systemic administration of cell-free exosomes generated by human MSCs (hMSCs) with treatment initiated 24 h post injury in a rat model of TBI significantly reduces the neuroinflammation (Zhang et al., 2015; Zhang Y. et al., 2016). In these two studies, activation of GFAP + astrocytes and CD68 + microglia/macrophages was significantly suppressed by exosomes compared to the liposome treatment control (Zhang et al., 2015; Zhang Y. et al., 2016). As the activated astrocytes and microglia play an important role in the initiation and progression of neuroinflammation following TBI by releasing inflammatory mediators, suppressing them by MSCs-derived exosomes can bring an anti-inflammatory effect which is just like that of MSC therapy in TBI animal models (Zhang et al., 2013). These studies indicate that MSCs-derived exosomes might have the potential to be a new novel strategy to treat neuroinflammation of TBI for their advantages including nanosize, low immunogenicity, ability to cross the BBB, capacity to efficiently transport a series of biological molecules and interact with plenty of targeted cells. Although above studies indicate that MSCs-derived exosomes have advantages and potential to be a novel treating strategy for neuroinflammation of TBI, the concrete cellular and molecular mechanism is still unclear. As the miRNAs involved in exosomes play a key role in modifying the phenotype and/or the physiology and modulating the cellular processes of the recipient cell (Cocucci and Meldolesi, 2015), they might be research targets in the future. In addition, previous studies have demonstrated that MSCs-derived exosomes can induce increased levels of anti-inflammatory cytokines and decreased levels of pro-inflammatory cytokines (Zhang et al., 2014), inhibit macrophage activation by suppressing Toll-like receptor signaling (Phinney et al., 2015) and inhibit hypoxic inflammation by suppressing pro-proliferative pathways such as STAT3 phosphorylation (Lee et al., 2012). These studies might provide some references and enlightenments for future researches about the molecular mechanisms and signal pathways of the reducing neuroinflammation effect in TBI conducted by MSCs-derived exosomes. What is more, the advantages of MSCs-derived exosomes may make them to be a potentially ideal drug delivery vehicle to treat neuroinflammation of TBI. The idea that exosomes can be taken as a tool to deliver therapeutic agents to treat inflammatory conditions was first demonstrated by Sun et al. (2010). The study showed that exosomes could carry and transmit curcumin which in turn enhanced their anti-inflammatory effects including increased stability and bioavailability of the complex and improved survival rate in lipopolysaccharide (LPS)-induced septicemia (Sun et al., 2010). Subsequently, one study clearly indicates that anti-inflammatory agents like curcumin or JSI124 can be effectively delivered to the brain by exosomes without observable side effects and alleviate LPS-induced brain inflammation in mice (Zhuang et al., 2011).

### MSCs-Derived Exosomes and the Neurogenesis of TBI

Following TBI, the primary injury initiatively induces irreversible brain damage and the secondary injury profoundly affects the process of the injury and clinical prognosis. There is no effective treatment for TBI up to now, making exploring new strategies be an urgent need. Recently, the research on neurogenesis is developing and neurogenesis turns to be a hopeful strategy for the repairment and regeneration in the injured brain after TBI based on the finding that neural stem cells (NSCs) are proven to exist in the adult brain and are capable of proliferating and generating functional neurons after brain damage. It was believed that neurogenesis could not be continued in the adult brain until latest studies indicate that some areas of the adult brain contain NSCs and retain the ability to generate neurons and glial cells, especially the subventricular zone (SVZ) and dentate gyrus (DG) of the hippocampus (Lois and Alvarez-Buylla, 1993; Gage et al., 1998). The extent of adult neurogenesis can be influenced by many factors including those related to TBI. One recent review concludes that neurogenesis is enhanced following TBI and plays an important role in the spontaneous cognitive function recovery after brain injury, and strengthening or regulating this endogenic cellular reaction might be a hopeful direction for researchers to explore new therapeutic strategies for brain repairment and regeneration following brain injury (Sun, 2016). This review provides plenty of strategies such as manipulating transcriptional regulators, supplementing various types of growth factors and acting intervention on pharmacological pathways targeting diverse aspects of the endogenous neurogenic responses that influence adult neurogenesis following TBI (Sun, 2016). In addition, several latest studies indicated that MSCs effectively promoted neurogenesis in experimental models of TBI (Lu et al., 2001; Chopp and Li, 2002; Mahmood et al., 2004a,b; Li and Chopp, 2009; Nichols et al., 2013). However, the predominant mechanisms by which MSCs participate in neurogenesis after TBI are not their cell replacement effects but likely their secretion-based paracrine effects (Chopp and Li, 2002; Li and Chopp, 2009). MSCs-derived exosomes are one kind of the MSCs secretion-based paracrines. Newly studies demonstrated that MSCs-derived exosomes promoted neurogenesis in animal models of TBI (Zhang et al., 2015; Kim et al., 2016; Zhang Y. et al., 2016). In these studies, MSCs-derived exosomes treatment significantly increased the number of newborn neurons (double staining for BrdU and NeuN) detected in the granule layer of the DG compared to the liposome controls. However, the concrete cellular and molecular mechanism of this neurogenic process is still unclear.

At present, most of the researchers who concentrate on the study about neurogenesis of TBI spare their efforts to explore new strategy to enhance cell proliferation, survival of new neurons in the neurogenic niche harboring active adult neurogenesis. So, the relationship between exosomes and neurogenic niches needs to be discussed before we investigate the role of MSCs-derived exosomes in the neurogenesis process of TBI. Neurogenic niche mainly exists in two areas of the brain: the subgranular zone (SGZ) of the DG in the hippocampus and the SVZ of the lateral ventricles (LV). It is a unique and specialized microenvironment which is composed of a variety of cells including glial cells, NSC-neuron lineage cells and vascular cells. Therefore, an efficient and well-regulated cell-to-cell communication in the adult neurogenic niches acts a crucial role in the actional regulation of plasticity and homeostasis in the neurogenesis process. Previous studies have made important progress to investigate the cellular components in the adult neurogenic niches and to explore the potential mechanisms by which they separately or cooperatively contribute to modulate adult neurogenesis (Aimone et al., 2014; Bjornsson et al., 2015; Migaud et al., 2016). Recently, a novel type of intercellular messengers “exosomes” have been identified. Exosomes seem to be a distinct intercellular communication medium for they are involved in the transferring of proteins, lipids, mRNAs and miRNAs between cells and therefore have the ability to modify the function of target cells (Cocucci and Meldolesi, 2015). Although the physiological and pathological roles of exosomes in the adult neurogenic niches remain virtually unexplored currently, the concept that exosomes have the potential to be a primary component available for regulating the functions of adult neurogenic niches is supported by more and more indirect evidences as follows. First, most of the cells in central nervous system (CNS) including NSCs and these cells that make up and modulate the neurogenic niche can release and/or be targets of exosomes. Second, some biological molecules generated and released by niche cells have been verified to exist in exosomes in physiological or pathological circumstances. Third, exosomes can serve as messengers between neural cells, communicators between blood and CNS and modulators of several stem cell niches (Batiz et al., 2015). Based on these findings, the neurogenic niche might be a research target for the exploration of the mechanisms of promoting neurogenesis in TBI conducted by MSCs-derived exosomes in the near future. For example, we can explore the possible molecular components of specific exosome populations in the neurogenic niche by using proper TBI animal models with which the exosomes can be labeled through molecular biology techniques and can be subsequently validated in health control and TBI with the use of a panel of biomarkers.

In addition, it is well known that several steps of adult neurogenesis are mediated by various miRNAs, but the definite targeting cells and mechanisms through which miRNAs are transmitted to targeting cells in the neurogenic niche are actually unknown. As several miRNAs including miR-let7b, miR-9, miR-124, miR-125b, and miR-128 that involved in the neurogenic/angiogenic process are contained in exosomes secreted by neurogenic niche cells (Zhao et al., 2009, 2010; Pogue et al., 2010; Huang et al., 2013; Cernilogar et al., 2015; Santos et al., 2016; Ye et al., 2016), and the miRNAs contained in exosomes play key roles in modifying the phenotype and/or the physiology and in modulating the cellular processes of the recipient cells (Cocucci and Meldolesi, 2015), they can be research points as well. One encouraging outcome was obtained in a middle cerebral artery occlusion model, in which the authors have presented that the level of miR-133b in MSCs significantly elevated when they were exposed to ischemic cerebral extracts, and the miR-133b can be transmitted to neurons and astrocytes via exosomes and further promotes neurite outgrowth and brain function recovery (Xin et al., 2012, 2013). This proof-of-concept study not only demonstrates that MSCs interact with neurons and astrocytes and modulate neurite outgrowth by transmitting miRNAs (miR-133b) via exosomes for the first time, but also helps to partially explain the mechanisms of MSCs’ contribution to neurological recovery through the identification of MSCs-derived exosomes as transporters that transfer miR-133b to neurons and astrocytes after stroke. MSCs-derived exosomes delivery functional miRNAs such as miR-133b which promotes neurite outgrowth might show benefit in other neurological disorders such as TBI as well. In addition, other researches that focused on non-disease models *in vitro* also demonstrated that MSCs-derived exosomes had the potential to assist neuron differentiation by delivering miRNAs. One of these researches reported that miR-124 and miR-145 can be delivered to astrocytes and NPCs through the contact independent and exosome-dependent procedure that changes the gene expression of recipient neural cells (Lee et al., 2014). These studies might provide some methodology references and enlightenments for the exploration of the mechanisms of the promoting neurogenesis effects conducted by MSCs-derived exosomes in TBI.

### MSCs-Derived Exosomes and the Therapy of TBI

Traumatic brain injury is a leading cause of death and longterm disability such as behavioral, cognitive and motor deficits worldwide. At present, there are mainly two therapeutic strategies to treat TBI, one is a neuroprotective treatment that targets the injured brain with a focus on reducing or preventing secondary injury and neural cell death and reducing the lesion size, the other one is a neurorestorative treatment aiming to improve neurological recovery via acting on the whole CNS to promote neurovascular remodeling including angiogenesis, neurogenesis, oligodendrogenesis and dendrite or axon outgrowth (Xiong et al., 2013). For a long time, the major efforts of treatment for TBI were the developments of neuroprotective agents and more than 30 clinical trials for potential treatment of TBI have been initiated during the past 3 decades, but almost all Phase II/III TBI clinical trials have failed (Janowitz and Menon, 2010; Loane and Faden, 2010). These reports indicate that no effective treatments currently exist that can improve neurological outcome after TBI and exploring new therapeutic approaches to improve functional recovery after TBI is an urgent need.

Until a decade ago, some researches demonstrated that the limited ability to exhibit structural and functional plasticity of brain after injury may prove to be significantly related to functional recovery (Chopp et al., 2008; Hall and Traystman, 2009; Xiong et al., 2013). Later, subsequent preclinical studies indicated that restorative treatments targeting multiple parenchymal cells including cerebral endothelial cells, NSCs and oligodendrocyte progenitor cells enhanced TBI-induced angiogenesis, neurogenesis, oligodendrogenesis and axonal sprouting respectively and further collectively improved neurological function after TBI (Xiong et al., 2010a,b). Recently, cell therapies using NPCs are promising for the treatment of brain injury (Jain, 2009). However, ethical considerations and other scientific problems restricted the clinical use of fetal tissues or ESCs. Then, researchers find that MSCs can be taken as a feasible source of stem cells for the cell replacement treatment as they are mesoderm-derived cells which mainly exist in UC blood, adult BM, peripheral blood and other organs and can differentiate into neuron cells (Greco and Rameshwar, 2007; Ho et al., 2008; Kassem and Abdallah, 2008). In the past decade, accumulating studies demonstrated that MSCs had promised to be an effective therapy for brain injury not only in experimental models of TBI (Lu et al., 2001; Chopp and Li, 2002; Mahmood et al., 2004a,b; Li and Chopp, 2009; Nichols et al., 2013), but also potentially in clinical practices (Zhang et al., 2008; Cox et al., 2011). While the predominant mechanisms by which MSCs participate in brain remodeling and functional recovery after TBI are likely related to their secretion-based paracrine effects rather than a cell replacement effect (Chopp and Li, 2002; Li and Chopp, 2009). MSCs-derived exosomes are one sort of the MSCs secretion-based paracrines characterized by without proliferation, easier preservation and transportation and less immunogenicity compared to MSCs (Lai et al., 2011). The diverse advantages of exosomes such as their bi-lipid membranes can keep their biological active constituents being stable which allows an easier storage and a longer half-life and shelf-life in patients, and their ability to cross the BBB and reach the brain parenchyma may make the transformation of MSC therapy from a cell-based one to a cell-free exosome-based one (Lai et al., 2013). What is more, the complex protein and genetic cargos of exosomes which have various biochemical potentials to participate in diverse cellular and biochemical processes can significantly contribute to the treatment of complicated diseases such as TBI. Some researchers even pointed out that the exosome might become a novel weapon for the treatment of TBI according to its nanosize, easy administration, ability to across the BBB, potential as a drug delivery tool and many other advantages (Alvarez-Erviti et al., 2011; Braccioli et al., 2014). Excitingly, latest studies demonstrated that intravenous administration of MSCs-derived exosomes significantly improved functional recovery, rescued pattern separation and spatial learning impairments, promoted neurovascular remodeling (angiogenesis and neurogenesis) and reduced neuroinflammation in TBI animal models (Zhang et al., 2015; Kim et al., 2016; Zhang Y. et al., 2016). Above studies indicate that MSCs-derived exosomes provide a potentially novel cell-free therapy for TBI. As for the mechanisms of the therapeutic effects of MSC-derived exosomes in TBI, though Zhang Y. et al. (2016) pointed out that the improved brain function recovery after treating TBI with exosomes derived from hMSCs had significant correlation with increased neurogenesis and angiogenesis and reduced neuroinflammation, the implicited cellular and molecular components and mechanisms needed to be explored in future studies.

In addition, as exosomes contain various miRNAs which play a key role in modifying the phenotype and/or the physiology and modulating the cellular processes of the recipient cell (Cocucci and Meldolesi, 2015), and miRNAs such as miR-21 could be potential therapeutic targets for interventions after TBI (Ge et al., 2014; Sandhir et al., 2014), the combination of miRNAs and MSC-derived exosomes might be a novel approach for the treatment of TBI. That is, MSCs-derived exosomes that carry and transfer their cargo such as miRNAs to parenchymal cells may mediate brain plasticity and improve functional recovery after TBI. Our conception might be partly supported by the studies which demonstrated that miR-133b, increased in MSCs exposed to ischemic cerebral extracts, was transferred to neurons and astrocytes via exosomes and promoted neurite outgrowth and brain function recovery in a middle cerebral artery occlusion animal model (Xin et al., 2012, 2013). Another development direction of the MSCs-derived exosomes treatment in TBI is their potential to act as a ‘ideal drug delivery vehicle’ on account of their desirable characteristics including low immunogenicity, capacity to efficiently transfer a variety of biological molecules and interact with a plenty of recipient cells, and significantly, their ability to manipulate personalized medicine for some diseases such as TBI. Exosomes can be operated *ex vivo* to carry not only therapeutic drugs but also short interfering RNAs (siRNAs) targeted specific genes in the brain (van den Boorn et al., 2011). One study indicated that exosomes generated from self-derived dendritic cells inhibited target gene expression in the brain via transporting siRNA to neurons, oligodendrocytes and microglia, and that exosomes loaded with siRNAs could be intravenous administrated and further silence the gene expression in CNS (Alvarez-Erviti et al., 2011). Above studies indicate that MSCs-derived exosomes can potentially act as a non-invasive intervention for the transportation of therapeutic agents into brain and further be applied in the treatment of TBI.

## Conclusion and Future Prospects

Exosomes are the smallest EVs that contain proteins, lipids, mRNAs and miRNAs and play an important role in the cell–cell communications. MSCs-derived exosomes have many advantages and promise to be a better therapeutic strategy for TBI compared to MSCs. Newly studies indicate that MSCs-derived exosomes improved functional recovery, promoted neurogenesis and reduced neuroinflammation in rats after TBI. Because the involved cellular and molecular mechanisms of the effects produced by MSCs-derived exosomes in TBI are unclear, some considerations are proposed in this review: (1) The miRNAs might be potential research targets to explore the reducing neuroinflammation and promoting neurogenesis effects produced by MSCs-derived exosomes. (2) The neurogenic niche might be a key research target to explore the promoting neurogenesis effects produced by MSCs-derived exosomes. Further, the combination of miRNAs and MSC-derived exosomes might be a novel approach for the treatment of TBI and MSC-derived exosomes have the potential to serve as a non-invasive intervention for successful delivery of therapeutic agents such as drugs and siRNAs to the brain and treat TBI.

Although the results of previous proof-of-concept studies are very encouraging, it is just a beginning for us to understand the potential of MSCs-derived exosomes as a viable therapeutic intervention for TBI. Several key points for the future exploration of the implicit involved cellular and molecular mechanisms of the effects produced by MSCs-derived exosomes in TBI and of the development of MSC-derived exosomes to treat TBI should be proposed, such as: (1) Alteration of exosomes cargos such as miRNAs in the neurogenic niches of TBI animal model and clinical patients, (2) Change of exosomes characteristics and their cargos such as miRNAs in the cerebrospinal fluid and peripheral blood of TBI model and patients, (3) Molecular constituents included in MSCs-derived exosomes such as miRNAs that promote angiogenesis and neurogenesis as well as reduce neuroinflammation after TBI, (4) Available approaches to engineer specific miRNAs into MSCs-derived exosomes, (5) Precise percentage of MSCs-derived exosomes that cross the BBB and finally reach the brain tissue, (6) Association between dose and effect of MSCs-derived exosomes in TBI, (7) Construction of standard MSCs source and culture conditions, (8) Exploration of effective methods to isolate and identify MSCs-derived exosomes from MSCs. Hence, much work should be conducted to take full advantages of MSCs-derived exosomes and support their potential to be a new strategy for the treatment of TBI in the future. We envisage that many of this work will be addressed by growing intense researches in the field of MSCs-derived exosomes and TBI through a yet unsuspected manner.

## Author Contributions

XH designed and guided the writing of this review, YxY and YY cooperatively wrote this review, XS, JH, and WB collected reference data for this review.

## Conflict of Interest Statement

The authors declare that the research was conducted in the absence of any commercial or financial relationships that could be construed as a potential conflict of interest.
